# Sequential SEM-EDS, PLM, and MRS Microanalysis of Individual Atmospheric Particles: A Useful Tool for Assigning Emission Sources

**DOI:** 10.3390/toxics9020037

**Published:** 2021-02-18

**Authors:** Francisco E. Longoria-Rodríguez, Lucy T. González, Yasmany Mancilla, Karim Acuña-Askar, Jesús Alejandro Arizpe-Zapata, Jessica González, Oxana V. Kharissova, Alberto Mendoza

**Affiliations:** 1 Centro de Investigación en Materiales Avanzados S.C. (CIMAV-Mty), Unidad Monterrey, Alianza Norte 202, Apodaca C.P. 66628, N.L., Mexico; francisco.longoria@cimav.edu.mx (F.E.L.-R.); alejandro.arizpe@cimav.edu.mx (J.A.A.-Z.); 2 Tecnologico de Monterrey, Escuela de Ingeniería y Ciencias, Ave. Eugenio Garza Sada 2501 Sur, Monterrey C.P. 64890, N.L., Mexico; y.mancilla@tec.mx; 3 Laboratorio de Biorremediación Ambiental, Facultad de Medicina, Universidad Autónoma de Nuevo León, Monterrey C.P. 64460, N.L., Mexico; karaskar@gmail.com; 4 Facultad de Salud Pública y Nutrición, Universidad Autónoma de Nuevo, Monterrey C.P. 64460, N.L., Mexico; argentinacrd@gmail.com; 5 Centro de Innovación, Investigación y Desarrollo en Ingeniería y Tecnología, Facultad de Fisicomatemáticas, Universidad Autónoma de Nuevo, San Nicolás de los Garza C.P. 66455, N.L., Mexico; okhariss@mail.ru

**Keywords:** sequential microanalysis, total suspended particles, polarized light microscopy, microRaman spectroscopy, scanning electron microscopy

## Abstract

In this work, the particulate matter (PM) from three different monitoring stations in the Monterrey Metropolitan Area in Mexico were investigated for their compositional, morphological, and optical properties. The main aim of the research was to decipher the different sources of the particles. The methodology involved the ex situ sequential analysis of individual particles by three analytical techniques: scanning electron microscopy-energy dispersive X-ray spectroscopy (SEM-EDS), polarized light microscopy (PLM), and micro-Raman spectroscopy (MRS). The microanalysis was performed on samples of total suspended particles. Different morphologies were observed for particles rich in the same element, including prismatic, spherical, spheroidal, and irregular morphologies. The sequential microanalysis by SEM-EDS/PLM/MRS revealed that Fe-rich particles with spherical and irregular morphologies were derived from anthopogenic sources, such as emissions from the metallurgical industry and the wear of automobile parts, respectively. In contrast, Fe-rich particles with prismatic morphologies were associated with natural sources. In relation to carbon (C), the methodology was able to distinguish between the C-rich particles that came from different anthopogenic sources—such as the burning of fossil fuels, biomass, or charcoal—and the metallurgical industry. The optical properties of the Si-rich particles depended, to a greater extent, on their chemical composition than on their morphology, which made it possible to quickly and accurately differentiate aluminosilicates from quartz. The methodology demonstrated in this study was useful for performing the speciation of the particles rich in different elements. This differentiation helped to assign their possible emission sources.

## 1. Introduction

Particulate matter (PM) encompasses a complex set of solid or liquid particles, or a combination of the two types, that are suspended in ambient air. Due to the great variety of emission sources, PM shows considerable variability in chemical composition, size, and morphology. It is well documented that prolonged exposure to PM can have adverse effects on human health [1,2,3,4,5]. In addition, recent studies that were carried out in several countries have identified links between contamination by PM and morbidity from the COVID-19 disease [6,7,8]. In recent years, the characterization of PM emissions and the identification of their sources have proved to be fundamental in determining control strategies for reducing emissions and risk exposures and for improving environmental public health policies that protect human health [9,10]. Various methodologies have been developed that allow for identification of emission source contributors to the presence of airborne PM, based on the chemical composition of the PM itself [11]. In particular, multivariate receptor modeling techniques are based on mass conservation, and the specific mathematical technique used to solve the mass conservation equations is subject to the available *a priori* information. Notable examples include the Chemical Mass Balance (CMB) model [12], which also requires knowledge on the number and chemical profiles of the sources, or the Positive Matrix Factorization (PMF) approach [13] that can be used if the number and nature of the sources are unknown. The identification of the PM sources obtained through these techniques, however, presents some uncertainties due to the nature of the data. In addition, numerical problems do arise that have an impact on interpreting the results from the model applications. CMB solutions can suffer from collinearity in the source profiles [14]; PMF is intrinsically based on an ill-posed problem where the experience of the analyst is fundamental in defining the number and nature of the main sources based on the obtained factors [15]. Additional information on the nature of the PM becomes relevant, as the mathematical models can have limitations in identifying the proper sources involved.

Most chemical characterization techniques used for emission source designations through receptor models are based on the outcome of a wide array of analyses on the bulk of particles. Recent studies have shown that an individual characterization of atmospheric particles can provide valuable information on their emission sources, formation processes, and reactivity [16,17]. Most of the techniques used to characterize individual particles do not require extensive sample pretreatment and are not destructive [18,19]. The scanning electron microscopy (SEM) with energy-dispersive X-ray spectroscopy (EDS) analysis is a nondestructive technique that can assess the chemistry of atmospheric PM and the processes that aerosols undergo in the atmosphere. The SEM-EDS is used to determine the elemental composition, morphology, and size of particles and has been proven to be helpful in differentiating particles based on their origins [20].

The SEM-EDS technique has been commonly used to characterize individual atmospheric particles and has been enhanced through the implementation of a computer-automated system (CC-SEM-EDS) that enables the characterization of many more particles with even greater accuracy and speed [21]. This technique has also been combined with statistical tools and computational methods (including Monte Carlo) for the analysis of atmospheric particles to identify their potential emission sources [22]. The use of SEM-EDS to investigate PM morphology has received increasing attention because the morphology of the particles appears related to their optical and compositional properties [23,24]. However, since the elemental analysis by SEM-EDS is semi-quantitative (i.e., under certain conditions, it generates errors close to 10%) and does not present information on the composition at the molecular level, it is necessary to complement it with other analytical methods, such as infrared microscopy or Raman spectroscopy. Micro-Raman spectroscopy (MRS) combines the analytical capabilities of Raman spectroscopy with the resolution of optical microscopy, thus providing the possibility to obtain the fingerprint spectra of each molecular species from individual particles and facilitating the detection of smaller groups of particulate species [25,26]. The characterization of environmental particles through MRS has been used to determine inorganic salts [27], biological materials [28], organosulfates [29], minerals [30], and carbonaceous materials, including black carbon (C) and brown C, among others [31].

Another technique used to characterize the individual particles is polarized light microscopy (PLM), which can examine particles with apparent diameters of up to 0.25 µm. Some optical properties, including color, refractive index, birefringence, and morphology, can be determined by PLM, which therefore represents a valuable supplementary technique in particle identification. Although PLM is a well-known technique, its application to the characterization of atmospheric particulate material has received little attention. Petean et al. (2017) analyzed PM_10_ and PM_2.5_ with PLM to identify phases such as quartz, kaolinite, calcite, muscovite, and goethite in the mineral fraction [32]. The biogenic fraction was also characterized by determining several species of pollen during late spring and early summer in Cluj-Napoca, Romania. In addition, Hindy et al. (2018) determined the mineralogical composition of urban dust collected in Cairo, Egypt, and identified species such as calcite, quartz, and gypsum, among others [33]. In Monza, Italy, Comite et al. (2020) used the PLM technique to characterize the damage to a landmark cathedral caused by exposure to polluted air [34].

In recent years, studies have used a combination of the aforementioned techniques to characterize ambient air PM and other environmental pollution materials, thus highlighting the significance and usefulness of these analytical tools in the analysis of environmental samples [35,36,37]. No studies, however, have applied the combined and sequential use of microanalysis techniques on the same particle. The current work presents a new analytical method for characterizing PM. The method comprises the sequential application of SEM-EDS, PLM, and MRS on the same particle to establish the relationships between morphology and the elemental and molecular composition of the particle. The information generated by this methodology is useful for assigning, with greater certainty, the probable sources of PM emissions.

## 2. Materials and Methods

### 2.1. Monitoring Stations and Total Suspended Particulates (TSP) Sampling

The Monterrey Metropolitan Area (MMA) is located in Northeastern Mexico, has an estimated population of 5 million people, and is the third-most populated metropolitan area in the country. Approximately 2 million vehicles, including automobiles, public transport buses, and heavy cargo trucks, commuted throughout the MMA in 2016 [38]. In addition, a large amount of industrial and commercial activity has eroded the ambient air quality such that the MMA has recently been considered one of the most polluted cities in Mexico [39]. Typical climate conditions are cold and dry weather in winter, with an average temperature ranging from 14.6–28.4 °C and an average annual rainfall of 583.2 mm [40].

Even though PM_2.5_ levels are of concern in the MMA, high levels of coarse PM are a major focus of interest for this region. According to 2018 data [41], annual PM_10_ averages varied between 37 μg/m^3^ and 74 μg/m^3^ among the 13 different air quality monitoring stations operating in the MMA; all except one exceeded the standard of 40 μg/m^3^. The maximum 24 h average PM_10_ concentration reported at any given station was 204 μg/m^3^, while the lowest maximum 24 h average PM_10_ concentration was 112 μg/m^3^. All stations were in violation of the 24 h standard of 75 μg/m^3^. Past studies have demonstrated a high load of geological (+45%) and organic material (10–17%) in the coarse fraction of the PM found in the ambient air of the MMA [42]. However, since a large part of the industrial plants in the region have emissions whose main traces are also present in the resuspended powders (e.g., cement plants, ceramic plants, glass plants, clay-related plants, steel-mills, metal processing plants, open-pit mining of limestone, and crushed stone/gravel processing) [43], source apportionment studies may be misrepresenting the main source contributions in this semi-arid region, which is also influenced by other anthopogenic fugitive dust emissions (e.g., road-dust resuspension and dust resuspension from the construction industry) as well as natural emissions. Thus, the proposed sequential use of microanalysis techniques can be useful to provide additional evidence on the most probable emission sources impacting a given site.

**Figure 1:**
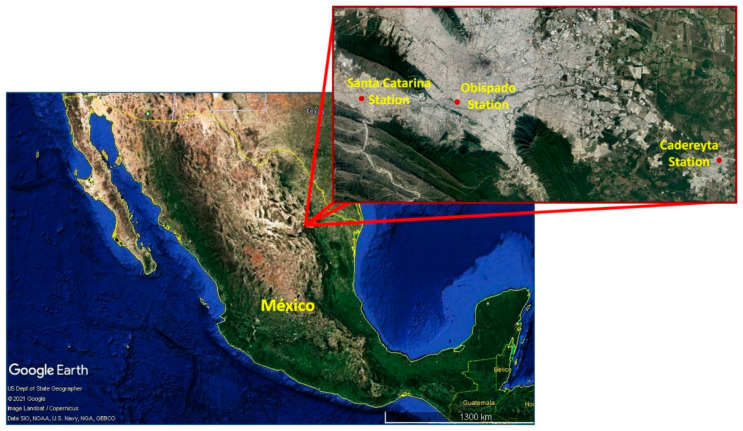
Monitoring stations in the Monterrey Metropolitan Area (MMA) where particulate matter (PM) sampling was conducted. Source: Google Earth

TSP were collected at three monitoring stations of the Integral Environmental Monitoring System (SIMA, by its initials in Spanish) of the MMA. The monitoring stations were selected based on the diversity of nearby industrial and commercial emissions to ascertain the potential pollution sources. Four samples were collected from each station during the winter period in December 2017. Figure 1 shows the locations of the selected monitoring stations.

One station was at Cadereyta (25°36′02.4″ N, 99°59′42.1″ W, 323 m above sea level [m.a.s.l.]), which is a relatively small town of 95,534 inhabitants. This is an important ambient air monitoring point because it is located 40 km southeast of Monterrey City and hosts a major refinery plant that produces a wide range of products [44]. The second monitoring station was located in Obispado (25°40′33.68″ N, 100°20′18.8″ W, 560 m.a.s.l.), which is characterized by a large amount of vehicular traffic and is the heart of Monterrey City, hosting schools, medical services, and commercial establishments, with little industrial activity. In addition, Obispado has been the site of important construction activities in recent years, including at the time of sample collection. The third sampling site was the Santa Catarina monitoring station (25°40′32.45″ N, 100°27′54.07″ W, 822 m.a.s.l.). This location is characterized by a wide range of intensive industrial activities and heavy freight traffic.

TSP samples were collected using a High Volume-Total Suspended Particulates sampler (model TE-310, TISH) over 24 h, with a mean airflow of 68 m^3^/h. The samples were collected at each monitoring station over consecutive days. A humidity chamber (<50%) set at 20–25 °C for 24 h was used to condition the glass fiber filters before and after the sampling procedures in accordance with the Compendium Method IO-2.1 [45].

### 2.2. Elemental Mapping of TSP Samples Subject to SEM-EDS and PLM

The morphology, elemental composition, and optical properties of the collected particles were analyzed using SEM-EDS and PLM techniques. To capture an overall perspective of the TSP, the samples collected from the three monitoring stations were mapped, and further individual analyses were performed on specific particles of interest. The collected atmospheric particles were mechanically removed (without using chemicals) from the glass fiber filters and placed on C tape. Low-resolution (<5000 X) electronic scanning was performed with a SEM (JEOL JSM-6010PLUS/LA) under low-vacuum conditions at 30 Pa using a backscatter electron (BSE) detector. For the elemental analyses, a JEOL EX94400T4L11 Dry SD detector was used and operated at 15 kV. The optical properties of the PM placed on the C tape were measured using an inverted reflected light Olympus^®^ GX51PL microscope. Micrographs were obtained using both an Olympus light polarizer and a GX-AN fixed analyzer. A digital Moticam^®^ 1080 supplied with a Complementary Metal-Oxide-Semiconductor (CMOS) sensor was used to obtain the images.

To complement the chemical mapping results, the crystalline phases in the PM were analyzed by X-ray diffraction (XRD). A representative amount of sample was removed from the filter and deposited onto a zero background holder made of amorphous silicon. The PM characterization was carried out with a PANalytical Empyrean X-ray diffractometer operated at 45 kV and 40 mA. The scans were performed in the 2Ɵ range from 5° to 90° with a step scan of 0.016° and 59 s per step. Structural refinements by the Rietveld method and crystalline phase identification were performed with the X’Pert High Score Plus software version 3.0.5 and the ICDD PDF+4 plus database (ICDD, International Center for Diffraction Data, Newtown Square, PA, USA).

### 2.3. SEM-EDS/PLM/MRS Characterization of Individual Microparticles

Sequential microanalysis (SMA) was applied to characterize the collected particles. The SMA methodology involved the ex-situ sequential analysis of individual particles by three analytical techniques (SEM-EDS/PLM/MRS). The particles were mechanically removed from the filters without tearing off the glass fibers and then placed onto a copper grid that was divided into quadrants. The SMA was carried out on composite samples from each monitoring station. The samples were first analyzed by SEM-EDS. Images were taken at low- and high-microscopy resolution. Low-resolution images were acquired using a JEOL scanning electron microscope, while high-resolution images were acquired using a FEI Nova NanoSEM™ (model 200) operated at 15 kV in high-vacuum mode with a BSE detector. Elemental analysis was made by an Oxford Instruments™ Inca X-Sight EDS detector.

**Figure 2:**
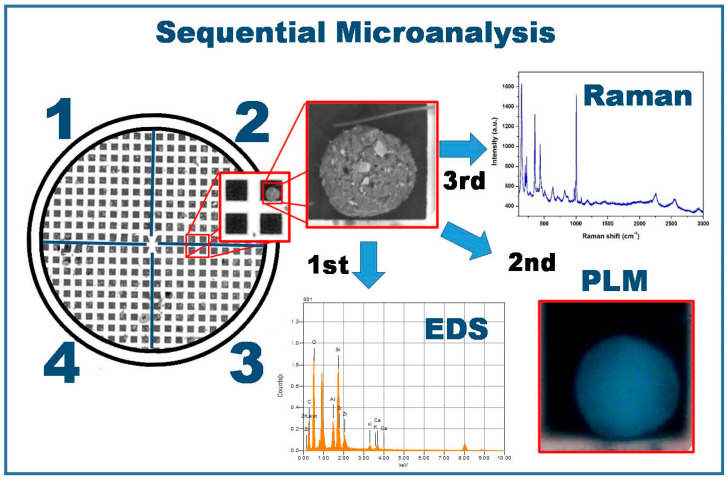
Description of the methodology used in the sequential microanalysis.

A polarized light microscope was used to determine the optical properties of the particles placed on the copper grid. The characteristics of the microscope are described in Section 2.2. A wide-range magnification objective lens (5X/0.1 BD, 50X/BD, and 100X/BD) was used to adequately visualize the particles of interest. Furthermore, the molecular composition of the particles was determined by MRS with a Horiba Scientific™ LabRam H Evolution Raman microscope at 40 mW. A 532 nm laser was directed onto each particle with the objective lens of an Olympus SLMPN 50X/0.35NA optical microscope. A Raman signal in the range of 5–20% of the beam intensity was achieved with a coupled charge detector at 220 K. The acquisition time was set at 5 s, and 15 spectra were added. The equipment calibration was performed with a silicon standard. Spectra were processed with the Horiba LabSpec 6 spectroscopy suite software. Figure 2 shows the sample preparation process used in the sequential microanalysis of the atmospheric particles.

## 3. Results and Discussion

### 3.1. Elemental Mapping of TSP Samples Subject to SEM-EDS and PLM

**Figure 3:**
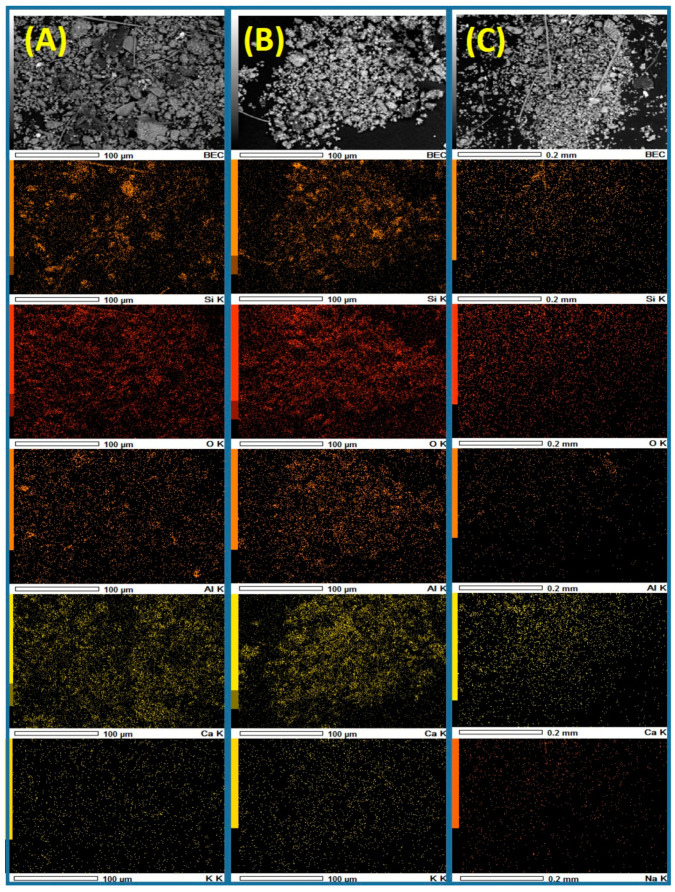
Chemical mapping of the particulate matter: we observed that the most abundant elements are Si, Ca, Al, and O for the Obispado (**A**) and Santa Catarina (**B**) areas, while for Cadereyta (**C**) they are Si, Al, and O.

Figure 3 shows the chemical mappings of the PM collected at the three monitoring stations. The particles showed an exceptionally varied morphology; many of them were spherical or spheroidal, while others presented cleavage and features typical of crystalline materials. Conglomerates of small particles with irregular morphologies were also observed. Several previous reports have indicated that anthopogenic particles usually present spherical or spheroidal morphologies because they are generated by vapor condensation [46], while those that arise from natural sources exhibit regular morphologies with angles, cleavages, and facets [47]. According to the intensity in the color corresponding to each element and the relative abundance (see Figure S1), the chemical mappings revealed that the most abundant elements in the collected particles were the following: Si, O, Ca, Al, K, and less proportion of detected Cl, Mg, S, and Fe. The presence of Si and Al may be associated with aluminosilicates. Ca and O might be associated with CaCO_3_ material, which is a highly predominant crystalline component of the PM in Monterrey’s atmosphere [48].

**Figure 4:**
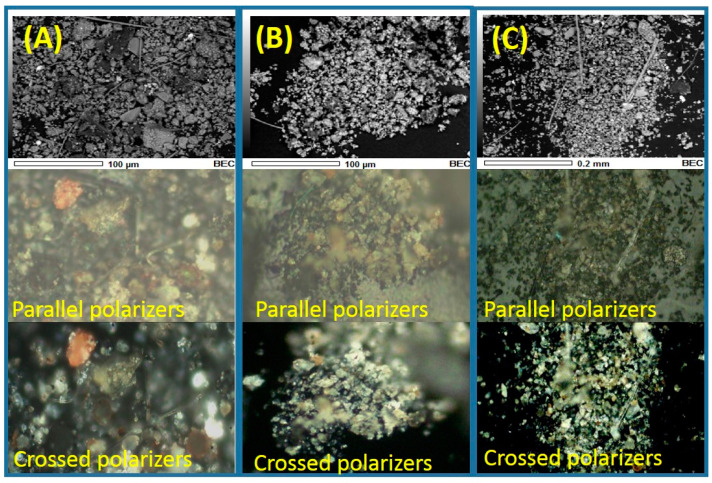
Micrographs and optical mappings with parallel and crossed polarizers obtained for the total suspended particle (TSP) collected in: (**A**) Obispado, (**B**) Santa Catarina, and (**C**) Cadereyta.

The samples were subsequently analyzed by PLM. The optical micrographs obtained by plane-polarized light are presented in Figure 4. We observe that the atmospheric particles show different colors like white (or colorless), gray, brown, yellow, light blue, green, and red. When the same particles were irradiated by cross-polarized light, the interference colors of most of the particles resembled their natural colors, and this can be attributed to a moderate to high degree of isotropy. In the case of the material collected at the Obispado station, the spherical and spheroidal particles rich in Si and Al acquired a white-colored tone at the hit of cross-polarized light, which may have been due to the presence of phyllosilicates [49]. At the Santa Catarina station, abundant white and colorless particles were recorded, possibly associated with CaCO_3_. Importantly, limestone mining is carried out intensively near this monitoring station, which could explain the abundance of this mineral in the collected PM. For the samples collected in Cadereyta, many yellow, white, and gray prismatic particles were observed in the micrographs. These particles may have been from soil resuspension because this station is located in a semi-rural area.

To corroborate the results above, the crystalline phases present in the PM were characterized by qualitative XRD and the quantitative Rietveld method. Table 1 shows the percentages of the crystalline phases present in the PM collected at the various monitoring stations of the MMA, and Figure S2 provides the obtained diffractograms. We observe that the most abundant phases in all the investigated samples are represented by CaCO_3_ (calcite), followed by SiO_2_ (quartz), aluminosilicates, CaMgC_2_O_6_ (dolomite), and CaSO_4_•2H_2_O. These results agree with the SEM-EDS and PLM mappings. Additionally, the highest percentage of CaCO_3_ was found in Santa Catarina, which could explain the PLM observations for these samples.

### 3.2. SEM-EDS/PLM/MRS Characterization of Individual Microparticles

To further study the relationship between the morphology, chemical composition of the particles, and their optical properties, an individual analysis was carried out sequentially combining SEM-EDS, PLM, and MRS. To locate the particles of interest, the grids were first mapped by SEM, sectioning them into quadrants. Once the particles were located on the grid, the image was captured, and point analysis was performed by EDS. Subsequently, the particles were characterized by PLM, followed by MRS. The particles were analyzed by MRS last because they can suffer damage from exposure to the laser that is used as an emission source in this technique. To obtain representative samples that included the greatest number of MMA emission sources, the characterization of the individual particles was carried out on composite samples from the three monitoring sites.

Overall, the PM in the MMA could be divided into three main groups: (i) spheroidal particles, (ii) particles with irregular morphology, and (iii) particles with angles and cleavages. A total of 10 particles from each group were analyzed, and the particles that represent the specific characteristics of each group are shown as an example.

**Figure 5:**
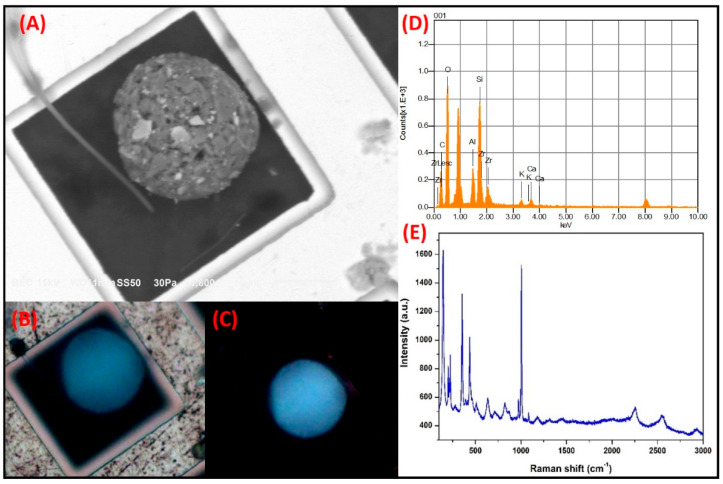
Sequential microanalysis (SMA) of a Si-rich particle showing a spheroidal morphology. (**A**) Electronic micrograph. (**B**) Polarized light micrograph with parallel polarizer, (**C**) crossed polarizer, (**D**) Elemental analysis by scanning electron microscopy-energy dispersive X-ray spectroscopy (SEM-EDS), and (**E**) Raman spectrum.

(i) Spheroidal particles. Based on their chemical composition, the group of spheroidal particles can be divided into the following three subgroups:

**Si-rich spheroidal particles**: Figure 5 shows the SMA of a spheroidal particle with a size of 25.2 µm. The micrograph clearly shows that it is formed by a conglomeration of smaller particles, some of which have facets and cleavages characteristic of crystalline materials. The EDS analysis revealed that the particle contained major constituents O, Si, and Zr with minor elements, including but not limited to Al, Ca and K. The C may be in the particle or may represent a surface coating. Optical micrographs obtained in the parallel and crossed polarization modes are shown in Figure 5B,C, respectively. The natural color of the particle seen in these micrographs resembles that of the characteristic third-order blue of zircon (ZrSiO_4_) [50]. Interestingly, no significant difference between the natural color and the interference color (with the crossed nicols) was found, an occurrence that may be related to the high isotropy level due to the spheroidal morphology of the particle. Isotropic opaque solids exhibit the same colors when the analyzer orientation is changed from a parallel to a crossed direction in relation to the position of the polarizer [51].

The Raman spectrum bands at 202.2 cm^−1^, 222.6 cm^−1^, 355 cm^−1^, 437.4 cm^−1^, 824.8 cm^−1^, 868.9 cm^−1^, 974.4 cm^−1^, and 1003 cm^−1^ can be associated with ZrSiO_4_ (Figure 5E), which supports the PLM analysis where the presence of this mineral was revealed. Additionally, the bands at 280.5 cm^−1^, 711.9 cm^−1^, and 1086.2 cm^−1^ can be associated with CaCO_3_. As mentioned above, particles with spheroidal morphologies are frequently associated with anthopogenic sources; therefore, the occurrence of these minerals could be related to the emissions of the construction industry or ceramics manufacturing. In this regard, studies carried out in the city of Castellón, Spain, revealed that particles with spherical morphology that contain Si and Zr are commonly released into the ambient air as part of the production of glazes and frits from the ceramics industry [52]. Based on the composition, morphology, and optical properties of the small particles, it can be assumed that the conglomerates arise from the grinding of raw materials in the aforementioned industries.

**Figure 6:**
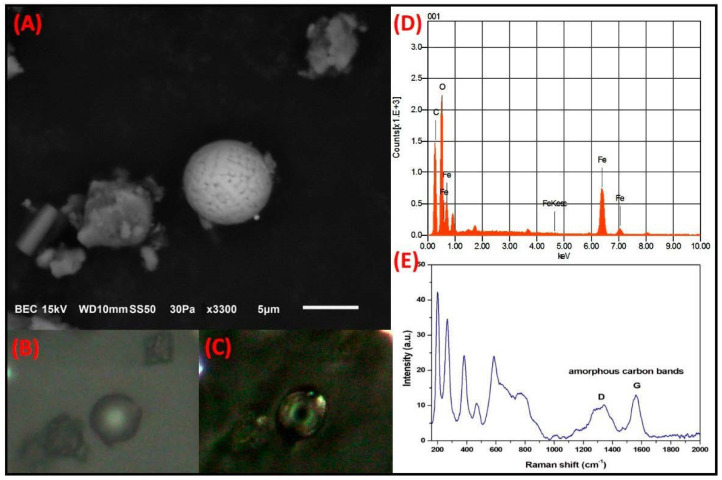
(**A**) Electron micrograph of a Fe-rich particle with spheroidal morphology and corrugated surface associated with anthopogenic sources. Optical micrograph with polarizer in (**B**) parallel and (**C**) crossed. (**D**) Elemental analysis by SEM-EDS and (**E**) Raman spectrum where the D and G bands of the carbonaceous material that coated the particle are observed.

**Fe-rich spheroidal particles.**Figure 6 shows another particle with spherical morphology and a corrugated surface texture, with a size of 8 µm. This particle has an elemental composition of Fe, O, and C. The PLM analysis revealed that the particle acquired a dark gray color when analyzed with the nicols in parallel; however, it presented birefringence with greenish tones under cross-polarization. In contrast, a previous report stated that when Fe-rich particles of mineral origin are analyzed under polarized light, the particle colors ranged from reddish-yellow tones to brown tones and did not show birefringence [53]. Therefore, it is likely that this spheroidal particle may have been covered with deposits of carbonaceous material that gave rise to the observed colors and birefringence. Infrared and fluorescence microscopy would be required to further understand the relationship between the unexpected interference colors and the exact composition of the carbonaceous material, and our research group is currently conducting studies on carbonaceous particles using these techniques. The Raman spectrum revealed the characteristic bands of hematite (Fe_2_O_3_; 224.87 cm^−1^, 286.914 cm^−1^, 404.5 cm^−1^, 493.6 cm^−1^, 604.9 cm^−1^), as well as bands of amorphous C (D band at 1361.5 cm^−1^ and G band at 1576.5 cm^−1^). Particles with similar morphologies and compositions have been associated with emissions from the metal-mechanical and metallurgical industries [54]. Notably, the MMA is located in a region with a large metal-mechanical and metallurgical presence. Therefore, it is highly likely that the large number of industries in the MMA contribute to the atmospheric emission of particles having the aforementioned characteristics [55].

Several reports have indicated that C deposits on particle surfaces can interfere with particle characterization. Gonzalez et al. (2018) demonstrated that both the D and G bands of amorphous C were able to mask the Raman signals of Fe-rich particles under a low-intensity laser. However, the carbonaceous covering of the particles degraded as the beam intensity was increased, thus allowing bands associated with Fe_2_O_3_ to be detected from the particles [56]. Similarly, Worobiec et al. (2010) indicated that the predominance of bands in the Raman spectrum that belong to carbonaceous deposits on the particle surface becomes so strong that it is difficult to perform the correct analysis [57].

**Figure 7:**
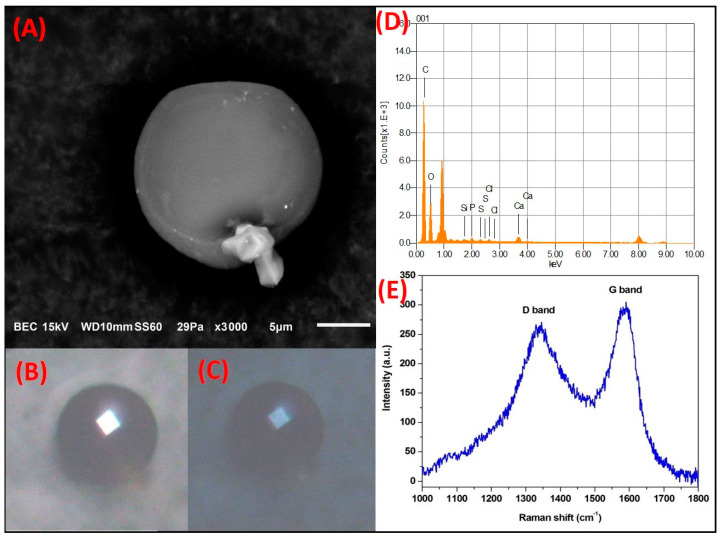
SMA of a spheroidal C-rich particle with a smooth surface. (**A**) SEM electron micrograph. Polarized light microscopy (PLM) micrograph with polarizer in (**B**) parallel and (**C**) crossed. (**D**) Elemental analysis by SEM-EDS and (**E**) Raman spectrum.

**C-rich spheroidal particles**. Particles with spheroidal morphology that were rich in C were also abundantly observed as part of the TSP in the MMA. Figure 7 shows the SMA of a spheroidal particle with a smooth surface and an approximate size of 17.7 µm. The particle was mainly composed of C. At lesser proportions, it contained O and trace amounts of S, Si, Ca, Cl and P. This composition and morphology are typical of black C particles. Several reports have related the physicochemical characteristics previously described with this type of particle [58,59]. Figure 7C,D show the optical micrographs obtained when the black C particle was exposed to polarized light. When the PLM analysis was carried out with the polarizers in parallel, the particle exhibited a natural black color. Similarly, when crossed nicols were used, the interference color did not differ considerably from the natural color. This would be expected because of the isotropy associated with the spheroidal morphology of the particle.

The Raman spectrum of the black C particle (Figure 7C) revealed the presence of two pronounced bands, characteristic of carbonaceous materials at ~1350 cm^−1^ and ~1560 cm^−1^, corresponding to the D and G bands, respectively [60]. The width and asymmetry of the peaks suggest the contribution of various modes of vibration from either the crystalline or molecular structures present on the particle. Several studies have suggested the contribution of five bands in the D and G peaks of C (G and D1–D4) and have proposed different methodologies to characterize carbonaceous materials based on spectroscopic parameters and curve-fitting procedures [57,61]. The deconvolution of the D and G bands for different carbonaceous particles collected in the MMA have been previously discussed in depth. Based on the results of previous analyses and a comparison with other studies, the most probable emission source of these particles is from burning fossil fuels such as gasoline and diesel [58].

**Figure 8:**
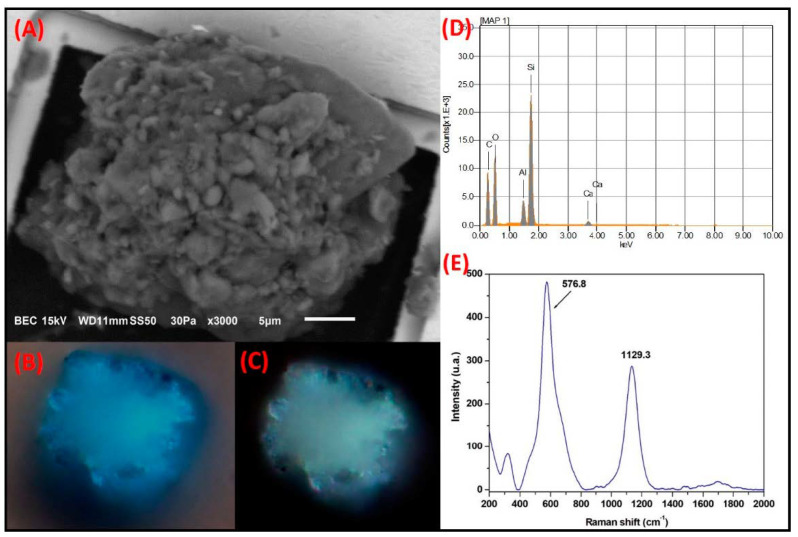
Si-rich particle conglomerate studied by SMA. (**A**) SEM electron micrograph. Typical third-order colors of silicates observed in optical micrographs using (**B**) parallel polarizer and (**C**) crossed. (**D**) Elemental composition by SEM-EDS and (**E**) Characteristic bands of silicates due to Raman modes present in these minerals.

Notably, most of the particles in this group presented a high level of isotropy that was associated with their spheroidal morphology. Moreover, both the natural and the interference color depended on the chemical composition of the particle.

(ii) Particles with irregular morphologies. This group can be divided into three subgroups:

**Si-rich irregular particles:**Figure 8 shows the SMA of a conglomerate of particles, most of which have a prismatic morphology and diameters smaller than 5 µm. The major elements in the particle were Si, C, O and, in a smaller proportion, Al and Ca.

The optical micrographs obtained with polarizers in parallel showed that the particle conglomerate presented a natural blue third-order color that is characteristic of some mineral aluminosilicates. Interestingly, when the micrograph was obtained with crossed polarizers, the particle conglomerate showed a blue-green third-order color that was likely due to a certain degree of anisotropy in the conglomerate itself. The natural color shown by the conglomerate was similar to that observed for the particle with spheroidal morphology examined in Figure 5 (ZrSiO_4_). Therefore, it is highly likely that the natural color of silicates is more influenced by the composition rather than the morphology, whereas the change of the interference color with respect to the natural color depends on the degree of anisotropy in the morphology of the particle.

The Raman spectrum showed bands at 330 cm^−1^, 576.8 cm^−1^, and 1129.3 cm^−1^, which are typical for aluminosilicates. Michaelian (1986) reported that the band at 330 cm^−1^ can be attributed to a combination of the vibration modes of the Si-O deformations and to the sheet vibrations of the octahedra in aluminosilicates, whereas the band at 576 cm^−1^ can be associated with the Si-O-Al bonds [62]. In addition, Yadav and Sigh (2015) attributed the band at 1129 cm^−1^ to the asymmetric vibrations of the Si-O bond [63]. Based on the morphology and the chemical characteristics of these particle conglomerates, it is probable that this group of particles is associated with the crustal material from soil resuspension. Aluminosilicate agglomerates similar to those observed herein have been reported by Heredia and Rodriguez (2016) in a study conducted in Aguascalientes, Mexico [64].

**Figure 9:**
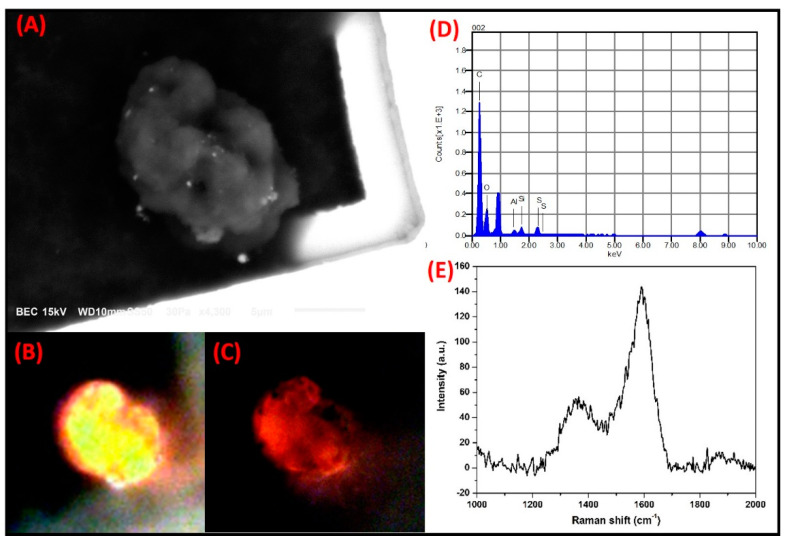
A high level of anisotropy was shown by a C-rich particle with irregular morphology when analyzed by SMA. (**A**) Electron micrograph. Optical micrographs with polarized light, using (**B**) the parallel and (**C**) crossed nicols. (**D**) Elemental composition by SEM-EDS and (**E**) Raman spectrum of the particle.

**C-rich irregular particles:** The micrograph in Figure 9 shows a particle with irregular morphology and a size of 14.4 µm. EDS analyses showed that the most abundant elements are C, O, and S, while Al and Si are very low (trace). When the C-rich particles were analyzed by PLM, their natural color through the polarizers in parallel was intense yellow, whereas their color shifted to intense red when cross polarizers were applied. This can be related to a high degree of anisotropy in the particle. According to Wu et al. (2015), particles with a similar composition and morphology have been associated with biomass burning in a study conducted in Shanxi, China [65]. Furthermore, like the particles in the current study, Micic et al. (2003) associated C-rich particles with an irregular morphology, and these particles were related to charcoal burning [66].

The results obtained by Raman spectroscopy confirmed the origin of these particles, since the D and G bands of amorphous C (1359 cm^−1^ and 1593 cm^−1^) showed a profile similar to that reported for the particles emitted by the burning of biomass as coal [67,68]. When this spectrum was compared with that obtained for the spheroidal C-rich particle, noticeable differences in the band profile were evident. These differences can be attributed to the diversity of the emission sources. Furthermore, charcoal grilling is quite common not only as a leisure activity for barbeque cooking but also as a commercial activity to prepare a wide variety of foods, and there is an increase in these activities throughout the winter [42,69,70].

**Figure 10:**
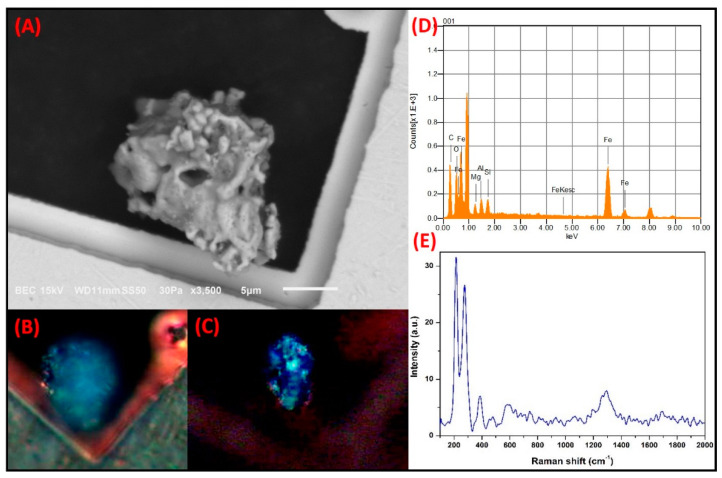
SMA study of a Fe-rich particle with irregular morphology associated with the wear of automobile parts. (**A**) SEM electron micrograph. Optical micrographs reveal the change in (**B**) natural color and (**C**) interference due to anisotropy in the particle microstructure. (**D**) Elemental analysis by SEM-EDS. (**E**) Raman modes of the hematite obtained by MRS.

**Figure 11:**
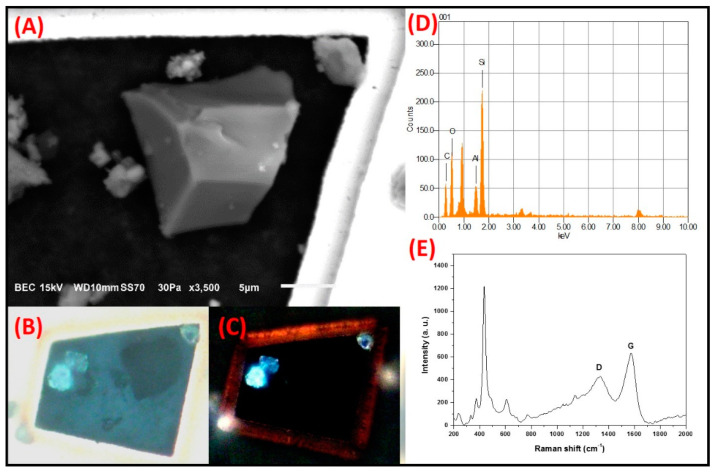
Prismatic Si-rich particles studied by SMA. (**A**) Electron micrograph. The analysis by PLM demonstrates the high level of isotropy in the particle because there is no variation in its (**B**) natural color and (**C**) interference. (**D**) Elemental analysis by SEM-EDS. (**E**) Raman spectrum showing bands associated with quartz.

**Fe-rich irregular particles:** Particles with irregular morphologies that were rich in Fe were also observed. Figure 10 shows the micrograph of a particle of this type with a size of 16.6 µm. The particles were mainly comprised of Fe, C, and O, with lower amounts of Al, Mg, and Si. The PLM analysis using the polarizers in parallel showed that the particle acquired different shades of third-order natural color that varied from green to blue. When it was analyzed with the crossed polarizers, the particle showed a slight change to second-order blue. The change in the natural and interference colors could be associated with the irregular morphology of the particle, which produced a high degree of anisotropy. It is possible that the blue color exhibited by these types of particles may be caused by the presence of Si and Al impurities. The Raman spectrum revealed bands at 218.77 cm^−1^ and 1295.15 cm^−1^, both of which coincide with the Raman modes of hematite [71]. The morphology and the elemental or molecular composition of this type of particle indicate that it is related to the material arising from the wear of automobile brakes and tires. Thorpe and Harrison (2008) reported that some chemical elements, including Fe, Al, and Mg, were components of particles that arose from brake wear [72], whereas Gonet and Maher (2019) established that hematite is one of the main compounds found in particles with an irregular morphology that are emitted from the wear of automobile parts [73].

(iii) Particles with facets and cleavages: This group contains particles that have faceted morphologies and cleavages that are typical of crystalline materials. This group can be divided into the following four subgroups:

**Si-rich prismatic particles:**Figure 11 shows the SMA of a particle with a size of 13 µm and a prismatic morphology characteristic of materials with long-range atomic ordering. The most abundant elements are Si, O, and C, while Al is much lower. Therefore, the particle could be characterized as a quartz particle. The optical micrographs obtained with the parallel and the crossed nicols revealed that the particle did not show any color in either case. It was thus assumed that the black color could be due to the structure of the particle presenting a certain degree of symmetry. Most quartz particles have a first-order color ranging from black and gray to white, and it has been confirmed that the low birefringence of quartz does not allow the particles to acquire second-order color [51]. The color exhibited by the prismatic particle differed markedly from that of the spherical and irregular silicon-rich particles. This might be related to differences in their composition, since spherical and irregular silicon-rich particles would correspond to zirconium and aluminum silicates, respectively, while those with a prismatic morphology would correspond to quartz particles. These results reveal that in Si-rich particles, the optical reflection properties of polarized light depend to a greater extent on the particle composition rather than on the morphology.

In the Raman spectrum, the bands at 233 cm^−1^, 1,357 cm^−1^, 1,544 cm^−1^, 1,636 cm^−1^, and 771 cm^−1^ match those previously reported for quartz. Importantly, the results obtained in this investigation through XRD revealed that one of the major phases in PM corresponds to quartz species (Table 1). The morphology and composition suggest that the particle can be associated with natural sources, including the abrasion and resuspension of crustal material [47]. Furthermore, it is known that when quartz becomes crushed, the resulting particles have irregular angular fragments with sharp edges, resembling the observations of this particle. Therefore, the ceramic and construction industries cannot be ruled out as possible emission sources of quartz particles.

**Figure 12:**
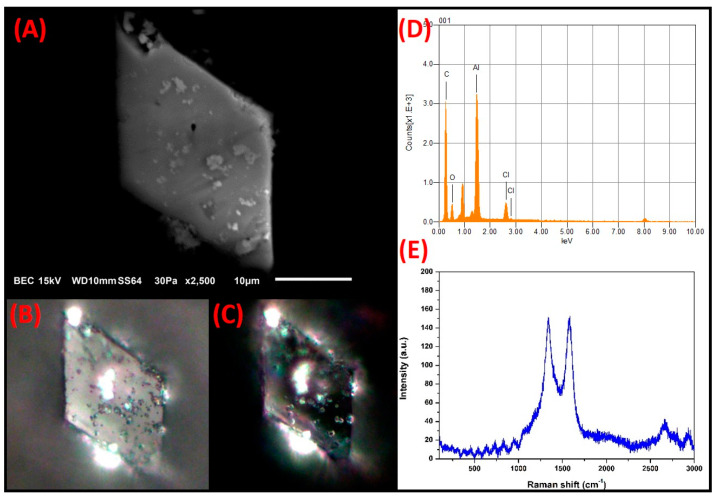
(**A**) Electron micrograph of a rhombic C-rich particle. Optical micrographs of polarized light with the nicols in: (**B**) parallel and (**C**) crossed. (**D**) Elemental analysis by SEM-EDS. (**E**) Raman spectrum.

**C-rich prismatic particles:** The micrograph of a particle with isometric rhombic morphology, well-defined facets, and a size of 21.58 µm × 38.3 µm is presented in Figure 12. The particle contained major elements O, Al, C, and minor Cl. In the optical micrograph obtained from the polarized light and parallel nicols analysis, unlike black C (which is bluish-black), the rhombic particle acquired a natural gray color. This gray color could be associated with the different chemical compositions of the particles. This is because black C particles contain elements associated with organic materials, whereas this rhombic particle is composed of elements associated with inorganic materials. The crossed polarization analysis revealed a change in the tone of the first-order gray color, which could be associated with a slight level of anisotropy present in the C-rich particle. The colors exhibited by the prismatic particle resembled those reported for carbonaceous materials from mineral C burning or carbide formation [74].

The Raman spectrum showed D (1337 cm^−1^) and G (1577 cm^−1^) bands of the functionalized C. Considerable differences in the band profiles were observed when the Raman spectra of this particle were compared with those obtained from the C-rich spheroidal particle (black C). These differences could be because the particles came from different emission sources. As mentioned above, numerous studies have linked spherical C-rich particles to emissions from diesel burning, and a characteristic profile has been established for both the D and G bands of their Raman spectrum. Moreover, the Raman spectrum of the C-rich rhombic particle has a profile that resembles that reported for C emissions from the metallurgical industry (carbides). This finding is consistent with the PLM results for this particle [75,76].

**Figure 13:**
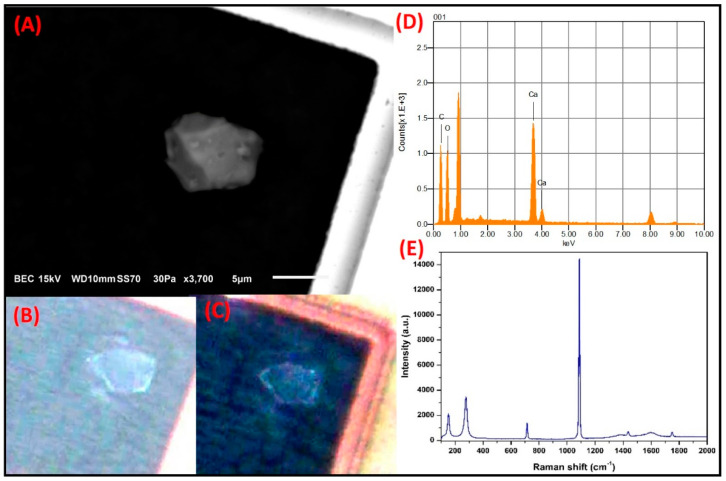
(**A**) Electron micrograph of a prismatic particle associated with CaCO3 from soil resuspension. (**B**) Natural color and (**C**) interference obtained by PLM. (**D**) Elemental analysis by SEM-EDS. (**E**) Bands related to the Raman modes of calcite.

**Ca-rich prismatic particles:**Figure 13 shows the SMA of a particle with a size of 6.5 µm × 8.6 µm and a prismatic morphology with facets typical of crystalline material. The particle contains Ca, O, and C. The PLM analysis revealed that when the nicols were used in parallel, the particle was colorless, whereas when the crossed polarizers were applied, the color shifted to blue. These results are consistent with the optical properties reported for calcite [77]. In the spectrum obtained by MRS, the bands observed at 152.9 cm^−1^, 278 cm^−1^, 714.4 cm^−1^, and 1087.6 cm^−1^ were also associated with CaCO_3_. Due to the morphology and composition, this particle type can be related to crustal material arising from soil resuspension; however, because of its size, its association with emissions from construction industry crushing and milling processes cannot be ruled out. This result is in agreement with the SEM-EDS elemental mapping, PLM, and XRD results (Table 1). In addition, calcite was one of the most abundant minerals in the PM, and no Ca-rich particles with other types of morphologies were found, thus strongly suggesting that their emission may be from crustal origin alone.

**Figure 14:**
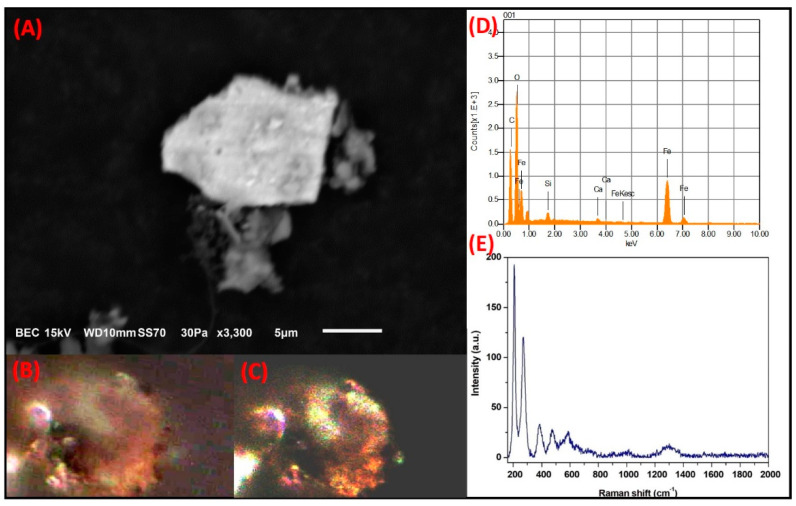
SMA study of a hematite particle possibly related to the crustal material of the MMA. (**A**) SEM electron micrograph. (**B**) Natural color and (**C**) interference typical of hematite mineral. (**D**) Elemental analysis by SEM-EDS. (**E**) Characteristic Raman spectrum of hematite by MRS.

**Fe-rich prismatic particles:** Another type of particle showing typical characteristics of the crystalline state corresponds to those rich in iron. Figure 14 shows the SMA of a particle with a size of 12.8 µm and a composition consisting of Fe, O and C with trace levels of Si and Ca. The natural and interference colors shown by this particle resembled the reddish colors reported by Iglesias et al. (2018) for hematite [78]. The following bands in the Raman spectrum were associated with hematite: 207.9 cm^−1^, 385 cm^−1^, 474.2 cm^−1^, and 585.6 cm^−1^. The origin of this particle subgroup could be related to natural sources because the morphology, composition, and optical properties coincided with those previously reported for this source type. The XRD analysis showed that hematite constituted one of the minority phases in the collected PM and may also be associated with materials from the Earth’s crust. In a study carried out in Mexico City using SEM-EDS, Labrada et al. (2012) found Fe-rich prismatic particles, which, through further in-depth analysis, revealed an association with soil resuspension [79]. Moreover, Doughty and Hill (2020) characterized the PM in Washington, D.C. through an automated aerosol Raman spectrophotometer and reported hematite mineral particles associated with soil resuspension [80].

A comparison of these results with those obtained for Fe-rich particles with spherical and irregular morphology (described in Section 1 and Section 2) shows that SMA can discriminate Fe-rich particles of natural origin from those deriving from anthopogenic processes.

## 4. Conclusions

The sequential microanalysis applied to TSP collected in the MMA has proved very effective in establishing the relationships between the morphology, chemical composition, and optical properties of the particles, which are of great importance in identifying the emission sources.

Based on their morphology, the particles collected from the three monitoring stations were assembled into three large groups, spheroidal, prismatic, and irregular, which were further subgrouped according to their chemical composition. Through the sequential study of the particles by SEM-EDS, PLM, and Raman spectroscopy, it was possible to clearly differentiate the C-rich particles that resulted from the burning of fossil fuels, biomass, or charcoal and those derived from the metallurgical industry. Similarly, the SMA helped to distinguish silicates originating from natural sources from those derived from anthopogenic sources, such as construction and ceramic industries. The speciation of the Fe-rich particles revealed that they were emitted by various sources, including the metallurgical industry, wear of automobile parts, and crustal material. In addition, deposits of carbonaceous material were observed on the Fe-rich spherical particles, which caused birefringence. The results obtained in this study showed that both morphology and chemical composition may influence the optical properties of particles. As a matter of fact, we observed that the natural and the interference color of the spheroidal and irregular particles depend on the chemical composition, while in the prismatic particles, the morphology may influence the optical properties.

The methodology used in this investigation was able to produce complete and detailed information at the micro and nano levels for the studied material. Therefore, its application may be feasible in other disciplines, such as the materials and forensic sciences and also the biomedical field.

## Figures and Tables

**Table 1:** Contents of the most important crystalline mineral phases present in the TSP from the three monitoring stations, obtained by X-ray Powder Diffractometry.

% Phases	Monitoring Station		
	Obispado	Santa Catarina	Cadereyta
Calcite	72.3 ± 1.6	77.9 ± 2.1	71 ± 1.9
Quartz	11.7 ± 1.2	10.3 ± 1.6	15.0 ± 1.3
Gypsum	11.1 ± 0.9	7.3 ± 1.1	10.4 ± 0.8
Aluminosilicates	3.0 ± 0.8	3.2 ± 1.0	1.7 ± 0.7
Hematite	1.8 ± 0.4	1.3 ± 0.7	1.9 ± 0.6

## Data Availability

All data generated or analyzed during this study are included in this published article.

## Supplementary Materials

The following are available online at https://www.mdpi.com/2305-6304/9/2/37/s1, Figure S1. Spectra and semi-quantitative EDS analyses obtained from particulate matter collect at (A) Obispado, (B) Santa Catarina, and (C) Cadereyta monitoring stations. Figure S2. Diffractograms obtained from the XRD analyses on particulate matter collected at (A) Obispado, (B) Santa Catarina and (C) Cadereyta monitoring stations.

## References

[1] Xing Y.-F., Xu Y.-H., Shi M.-H., Lian Y.-X. (2016). The impact of PM2.5 on the human respiratory system. J. Thorac. Dis..

[2] Grahame T.J., Klemm R., Schlesinger R.B. (2014). Public health and components of particulate matter: The changing assessment of black carbon. J. Air Waste Manag. Assoc..

[3] Betha R., Behera S.N., Balasubramanian R. (2014). 2013 Southeast Asian Smoke Haze: Fractionation of Particulate-Bound Elements and Associated Health Risk. Environ. Sci. Technol..

[4] Ostro B., Broadwin R., Green S., Feng W.-Y., Lipsett M. (2006). Fine Particulate Air Pollution and Mortality in Nine California Counties: Results from CALFINE. Environ. Health Perspect..

[5] Samoli E., Analitis A., Touloumi G., Schwartz J., Anderson H.R., Sunyer J., Bisanti L., Zmirou D., Vonk J.M., Pek-kanen J. (2005). Estimating the exposure-response relationships between particulate matter and mortality within the AP-HEA multicity project. Environ. Health Perspect..

[6] López-Feldman A., Heres D., Marquez-Padilla F. (2021). Air pollution exposure and COVID-19: A look at mortality in Mexico City using individual-level data. Sci. Total Environ..

[7] Travaglio M., Yu Y., Popovic R., Selley L., Leal N.S., Martins L.M. (2021). Links between air pollution and COVID-19 in England. Environ. Pollut..

[8] Zoran M.A., Savastru R.S., Savastru D.M., Tautan M.N. (2020). Assessing the relationship between surface levels of PM2.5 and PM10 particulate matter impact on COVID-19 in Milan, Italy. Sci. Total Environ..

[9] Hopke P.K., Dai Q., Li L., Feng Y. (2020). Global review of recent source apportionments for airborne particulate matter. Sci. Total Environ..

[10] Boldo E., Linares C., Lumbreras J., Borge R., Narros A., García-Pérez J., Fernández-Navarro P., Pérez-Gómez B., Aragonés N., Ramis R. (2011). Health impact assessment of a reduction in ambient PM2.5 levels in Spain. Environ. Int..

[11] Hopke P.K. (2016). Review of receptor modeling methods for source apportionment. J. Air Waste Manag. Assoc..

[12] Coulter C.T. (2004). Users Manual. Office of Air Quality Planning & Standards.

[13] Paatero P. (1997). Least squares formulation of robust non-negative factor analysis. Chemom. Intell. Lab. Syst..

[14] Shi G.-L., Feng Y.-C., Zeng F., Li X., Zhang Y.-F., Wang Y.-Q., Zhu T. (2009). Use of a Nonnegative Constrained Principal Component Regression Chemical Mass Balance Model to Study the Contributions of Nearly Collinear Sources. Environ. Sci. Technol..

[15] Reff A., Eberly S.I., Bhave P.V. (2007). Receptor modeling of ambient particulate matter data using positive matrix factoriza-tion: Review of existing methods. J. Air Waste Manag. Assoc..

[16] Utsunomiya S., Jensen K.A., Keeler G.J., Ewing R.C. (2004). Direct Identification of Trace Metals in Fine and Ultrafine Particles in the Detroit Urban Atmosphere. Environ. Sci. Technol..

[17] Geng H., Cheng F., Ro C.U. (2011). Single-Particle Characterization of Atmospheric Aerosols Collected at Gosan, Korea, dur-ing the Asian Pacific Regional Aerosol Characterization Experiment Field Campaign Using Low-Z (Atomic Number) Particle Electron Probe X-ray Microanalysis. J. Air Waste Manag. Assoc..

[18] Zeb B.B., Alam K.K., Sorooshian A.A., Blaschke T., Ahmad I., Shahid I. (2018). On the Morphology and Composition of Particulate Matter in an Urban Environment. Aerosol Air Qual. Res..

[19] Ji Z., Dai R., Zhang Z. (2014). Characterization of fine particulate matter in ambient air by combining TEM and multiple spectroscopic techniques – NMR, FTIR and Raman spectroscopy. Environ. Sci. Process. Impacts.

[20] Salma I., Maenhaut W., Zemplén-Papp É., Záray G. (2001). Comprehensive characterisation of atmospheric aerosols in Buda-pest, Hungary: Physicochemical properties of inorganic species. Atmos. Environ..

[21] Casuccio G.S., Schlaegle S.F., Lersch T.L., Huffman G.P., Chen Y., Shah N. (2004). Measurement of fine particulate matter using electron microscopy techniques. Fuel Process. Technol..

[22] Gokhale S., Patil R. (2010). Uncertainty in modelling PM10 and PM2.5 at a non-signalized traffic roundabout. Atmos. Pollut. Res..

[23] Adachi K., Chung S.H., Buseck P.R. (2010). Shapes of soot aerosol particles and implications for their effects on climate. J. Geophys. Res. Space Phys..

[24] Ghio A.J., Devlin R.B. (2001). Inflammatory Lung Injury after Bronchial Instillation of Air Pollution Particles. Am. J. Respir. Crit. Care Med..

[25] Craig R.L., Bondy A.L., Ault A.P., Craig R.L., Bondy A.L., Computer- A.P.A. (2017). Computer-controlled Raman microspec-troscopy (CC-Raman): A method for the rapid characterization of individual atmospheric aerosol particles. Aerosol Sci. Technol..

[26] Doughty D.C., Hill S.C. (2020). Raman spectra of atmospheric particles measured in Maryland, USA over 22.5 h using an automated aerosol Raman spectrometer. J. Quant. Spectrosc. Radiat. Transf..

[27] Jentzsch P.V., Kampe B., Ciobotă V., Rösch P., Popp J. (2013). Inorganic salts in atmospheric particulate matter: Raman spectroscopy as an analytical tool. Spectrochim. Acta Part A Mol. Biomol. Spectrosc..

[28] Ghosal S., Macher J.M., Ahmed K. (2012). Raman Microspectroscopy-Based Identification of Individual Fungal Spores as Poten-tial Indicators of Indoor Contamination and Moisture-Related Building Damage. Environ. Sci. Technol..

[29] Bondy A.L., Craig R.L., Zhang Z., Gold A., Surratt J.D., Ault A.P. (2017). Isoprene-Derived Organosulfates: Vibrational Mode Analysis by Raman Spectroscopy, Acidity-Dependent Spectral Modes, and Observation in Individual Atmospheric Particles. J. Phys. Chem. A.

[30] Sobanska S., Hwang H., Choël M., Jung H.-J., Eom H.-J., Kim H., Barbillat J., Ro C.-U. (2012). Investigation of the Chemical Mixing State of Individual Asian Dust Particles by the Combined Use of Electron Probe X-ray Microanalysis and Raman Microspectrometry. Anal. Chem..

[31] Tóth Á., Hoffer A., Pósfai M., Ajtai T., Kónya Z., Blazsó M., Czégény Z., Kiss G., Bozóki Z., Gelencsér A. (2018). Chemical characterization of laboratory-generated tar ball particles. Atmos. Chem. Phys..

[32] Petean I., Mocanu A., Păltinean G.A., Ţărcan R., Muntean D.F., Mureşan L., Arghir G., Tomoaia-Cotişel M. (2017). Physi-co-chemical study concerning atmospheric particulate matter hazard. Stud. Univ. Babes-Bolyai Chem..

[33] Hindy K.T., Baghdady A.R., Howari F.M., Abdelmaksoud A.S. (2018). A Qualitative Study of Airborne Minerals and Associated Organic Compounds in Southeast of Cairo, Egypt. Int. J. Environ. Res. Public Health.

[34] Comite V., Pozo-Antonio J.S., Cardell C., Randazzo L., La Russa M.F., Fermo P. (2020). A multi-analytical approach for the characterization of black crusts on the facade of an historical cathedral. Microchem. J..

[35] Morillas H., Marcaida I., García-Florentino C., Maguregui M., Arana G., Madariaga J.M. (2018). Micro-Raman and SEM-EDS analyses to evaluate the nature of salt clusters present in secondary marine aerosol. Sci. Total Environ..

[36] Fermo P., Mearini A., Bonomi R., Arrighetti E., Comite V. (2020). An integrated analytical approach for the characterization of repainted wooden statues dated to the fifteenth century. Microchem. J..

[37] Fermo P., Comite V., Ciantelli C., Sardella A., Bonazza A. (2020). A multi-analytical approach to study the chemical composi-tion of total suspended particulate matter (TSP) to assess the impact on urban monumental heritage in Florence. Sci. Total Environ..

[38] (2017). INEGI/Instituto Nacional de Estadística y Geografía Anuario estadístico y geográfico de Nuevo León 2017. Gob. Del Estado Nuevo León.

[39] Green J., Sánchez S. Air Quality in Latin America: An Overview—2012 Edition. Clean Air Institute. https://www.yumpu.com/en/document/view/41258091/air-quality-in-latin-america-an-overview-clean-air-institute.

[40] (2019). Centro Mario Molina Análisis de la Contaminación por PM2.5 en la Ciudad de Monterrey, Nuevo León, Enfocado a la Identificación de Medidas Estratégicas de Control. https://centromariomolina.org/wp-content/uploads/2019/05/3.-ResumenEjecutivo_CalidadAire_2018.pdf.

[41] (2018). Informe nacional de la calidad de aire México. Inst. Nac. Ecol. Cambio Climático.

[42] Mancilla Y., Paniagua I.Y.H., Mendoza A. (2019). Spatial differences in ambient coarse and fine particles in the Monterrey metropolitan area, Mexico: Implications for source contribution. J. Air Waste Manag. Assoc..

[43] de Monterrey S.D.S. Programa de Gestión para Mejorar la Calidad del Aire del Estado de Nuevo León ProAire 2016–2025. https://www.google.com.hk/url?sa=t&rct=j&q=&esrc=s&source=web&cd=&cad=rja&uact=8&ved=2ahUKEwjn9aOv9fLuAhXG7WEKHU8iDLgQFjABegQIAhAD&url=https%3A%2F%2Fwww.gob.mx%2Fsemarnat%2Facciones-y-programas%2Fprogramas-de-gestion-para-mejorar-la-calidad-del-aire&usg=AOvVaw1o4d1gjUfhPtqngbG8FYHh.

[44] INAFED, Plan Municipal de Desarrollo de Cadereyta 2018–2021 (2018). Periódico Of. del Estado. http://cadereyta.gob.mx/wp-content/uploads/2019/05/PLAN-MUNICIPAL-DE-DESARROLLO-2018-2021.pdf.

[45] U.S. EPA (1999). Environmental Protection Agency Methods Compendium Method IO-2.1.

[46] Li W., Shao L. (2009). Transmission electron microscopy study of aerosol particles from the brown hazes in northern China. J. Geophys. Res. Space Phys..

[47] Aragon-Piña A. (2011). ¿Cómo son las Partículas Atmosféricas Antropogénicas y Cuál es su Relación con los Diversos Tipos de Fuentes Contam-Inantes?.

[48] González L.T., Rodríguez F., Sánchez-Domínguez M., Leyva-Porras C., Silva-Vidaurri L., Acuna-Askar K., Kharisov B., Chiu J.V., Barbosa J.A. (2016). Chemical and morphological characterization of TSP and PM2.5 by SEM-EDS, XPS and XRD collected in the metropolitan area of Monterrey, Mexico. Atmos. Environ..

[49] Cabadas-Báez H.V., Sedov S., Jiménez-Álvarez S.D.P., Léonard D., Ancona-Aragón I.I., Hernández-Velázquez M.L. (2018). Soils as a source of raw materials for ancient ceramic production in the Maya region of Mexico: Micromorphological insight. Boletín Soc. Geológica Mex..

[50] Anthony J.W., Bideaux R.A., Bladh K.W., Nichols M.C. Handbook of Mineralogy.

[51] Delly J.G. (2007). Essentials of Polarized Light Microscopy and Ancillary Techniques.

[52] Pallarés S., Gómez E.T., Jordán M.M. (2019). Typological characterisation of mineral and combustion airborne particles in-doors in primary schools. Atmosphere.

[53] Iglesias J.C.A., Gomes O.D.F.M., Paciornik S. (2011). Automatic recognition of hematite grains under polarized reflected light microscopy through image analysis. Miner. Eng..

[54] González L.T., Rodríguez F.L., Sánchez-Domínguez M., Cavazos A., Leyva-Porras C., Silva-Vidaurri L.G., Askar K.A., Kharissov B.I., Chiu J.V., Barbosa J.A. (2017). Determination of trace metals in TSP and PM 2.5 materials collected in the Metropolitan Area of Monterrey, Mexico: A characterization study by XPS, ICP-AES and SEM-EDS. Atmos. Res..

[55] Centro Mario Molina (2019). PROYECTO: Propuestas Para el Desarrollo Sustentable de una Ciudad Mexicana. https://centromariomolina.org/wp-content/uploads/2019/05/2.-Resumen-Ejecutivo-Monterrey_218.pdf.

[56] González L.T., Longoria-Rodríguez F.E., Sánchez-Domínguez M., Leyva-Porras C., Acuña-Askar K., Kharissov B.I., Arizpe-Zapata A., Alfaro-Barbosa J.M. (2018). Seasonal variation and chemical composition of particulate matter: A study by XPS, ICP-AES and sequential microanalysis using Raman with SEM/EDS. J. Environ. Sci..

[57] Worobiec A., Potgieter-Vermaak S., Brooker A., Darchuk L., Stefaniak E., Grieken R. (2010). Van Interfaced SEM/EDX and micro-Raman Spectrometry for characterisation of heterogeneous environmental particles—Fundamental and practical challenges. Microchem. J..

[58] Longoria-Rodríguez F.E., González L.T., Mendoza A., Leyva-Porras C., Arizpe-Zapata A., Esneider-Alcalá M., Acu-ña-Askar K., Gaspar-Ramirez O., López-Ayala O., Alfaro-Barbosa J.M. (2020). Environmental Levels, Sources, and Can-cer Risk Assessment of PAHs Associated with PM2.5 and TSP in Monterrey Metropolitan Area. Arch. Environ. Contam. Toxicol..

[59] López-Ayala O., González-Hernández L.T., Alcantar-Rosales V.M., Elizarragaz-de la Rosa D., Heras-Ramírez M.E., Silva-Vidaurri L.G., Alfaro-Barbosa J.M., Gaspar-Ramírez O. (2019). Levels of polycyclic aromatic hydrocarbons associated with particulate matter in a highly urbanized and industrialized region in northeastern Mexico. Atmos. Pollut. Res..

[60] Sze S.K., Siddique N., Sloan J.J., Escribano R. (2001). Raman spectroscopic characterization of carbonaceous aerosols. Atmos. Environ..

[61] Ivleva N.P., McKeon U., Niessner R., Pöschl U. (2007). Raman Microspectroscopic Analysis of Size-Resolved Atmospheric Aerosol Particle Samples Collected with an ELPI: Soot, Humic-Like Substances, and Inorganic Compounds. Aerosol Sci. Technol..

[62] Michaelian K.H. (1986). The Raman spectrum of kaolinite #9 at 21 °C. Can. J. Chem..

[63] Yadav A.K., Singh P. (2015). A review of the structures of oxide glasses by Raman spectroscopy. RSC Adv..

[64] Rivera B.H., Rodriguez M.G. (2016). Characterization of Airborne Particles Collected from Car Engine Air Filters Using SEM and EDX Techniques. Int. J. Environ. Res. Public Health.

[65] Wu Z., Liu F., Fan W. (2015). Characteristics of PM10 and PM2.5 at Mount Wutai Buddhism Scenic Spot, Shanxi, China. Atmosphere.

[66] Micic M., Leblanc R.M., Markovic D., Stamatovic A., Vukelic N., Polic P. (2003). Atlas of the tropospheric aerosols from Bel-grade troposphere. Fresenius Environ. Bull..

[67] Inoue J., Yoshie A., Tanaka T., Onji T., Inoue Y. (2017). Disappearance and alteration process of charcoal fragments in cumula-tive soils studied using Raman spectroscopy. Geoderma.

[68] Smith M.W., Dallmeyer I., Johnson T.J., Brauer C.S., McEwen J.-S., Espinal J.F., Garcia-Perez M. (2016). Structural analysis of char by Raman spectroscopy: Improving band assignments through computational calculations from first principles. Carbon.

[69] Mancilla Y., Mendoza A., Fraser M.P., Herckes P. (2016). Organic composition and source apportionment of fine aerosol at Monterrey, Mexico, based on organic markers. Atmos. Chem. Phys. Discuss..

[70] Chacón D., Giner M., Vázquez M., Roe S., Maldonado J., Lindquist H., Strode B., Anderson R., Quiroz C., Scheiber J. (2010). Emisión de Gases de Efecto Invernadero en Nuevo León y Proyecciones de Referencia 1990–2025.

[71] Hanesch M. (2009). Raman spectroscopy of iron oxides and (oxy)hydroxides at low laser power and possible applications in environmental magnetic studies. Geophys. J. Int..

[72] Thorpe A., Harrison R.M. (2008). Sources and properties of non-exhaust particulate matter from road traffic: A review. Sci. Total Environ..

[73] Gonet T., Maher B.A. (2019). Airborne, Vehicle-Derived Fe-Bearing Nanoparticles in the Urban Environment: A Review. Environ. Sci. Technol..

[74] Walker D., Dasgupta R., Li J., Buono A. (2013). Nonstoichiometry and growth of some Fe carbides. Contrib. Miner. Pet..

[75] Urbonaite S., Hälldahl L., Svensson G. (2008). Raman spectroscopy studies of carbide derived carbons. Carbon.

[76] Rantitsch G., Bhattacharyya A., Schenk J., Lünsdorf N.K. (2014). Assessing the quality of metallurgical coke by Raman spec-troscopy. Int. J. Coal Geol..

[77] Osacky M., Geramian M., Dyar M.D., Sklute E.C., Valter M., Ivey D.G., Liu Q., Etsell T.H. (2013). Characterisation of petrologic end members of oil sands from the athabasca region, Alberta, Canada. Can. J. Chem. Eng..

[78] Iglesias J.C. (2018). Álvarez; Augusto, K.S.; Gomes, O.D.F.M.; Domingues, A.L.A.; Vieira, M.B.; Casagrande, C.; Paciornik, S. Automatic characterization of iron ore by digital microscopy and image analysis. J. Mater. Res. Technol..

[79] Labrada-Delgado G., Aragon-Pina A., Campos-Ramos A., Castro-Romero T., Amador-Munoz O., Villalobos-Pietrini R. (2012). Chemical and morphological characterization of PM2.5 collected during MILAGRO campaign using scanning electron microscopy. Atmos. Pollut. Res..

[80] Doughty D.C., Hill S.C. (2017). Journal of Quantitative Spectroscopy & Radiative Transfer Automated aerosol Raman spec-trometer for semi-continuous sampling of atmospheric aerosol. J. Quant. Spectrosc. Radiat. Transf..

