# Supplementary material for: Sequential SEM-EDS, PLM, and MRS Microanalysis of Individual Atmospheric Particles: A Useful Tool for Assigning Emission Sources
Source: Toxics. 2021 Feb 18;9(2):37. doi: 10.3390/toxics9020037 (PMC7922855; doi:10.3390/toxics9020037)
Supplement: Supplementary file 1 [file toxics-09-00037-s001.pdf]

toxics

Supplementary Materials: Sequential SEM-EDS, PLM, and MRS Microanalysis of Individual Atmospheric Particles: A Useful Tool for Assigning Emission Sources

Francisco E. Longoria-Rodríguez, Lucy T. González, Yasmany Mancilla, Karim Acuña-Askar, Jesús Alejandro Arizpe-Zapata, Jessica González, Oxana V. Kharissova and Alberto Mendoza

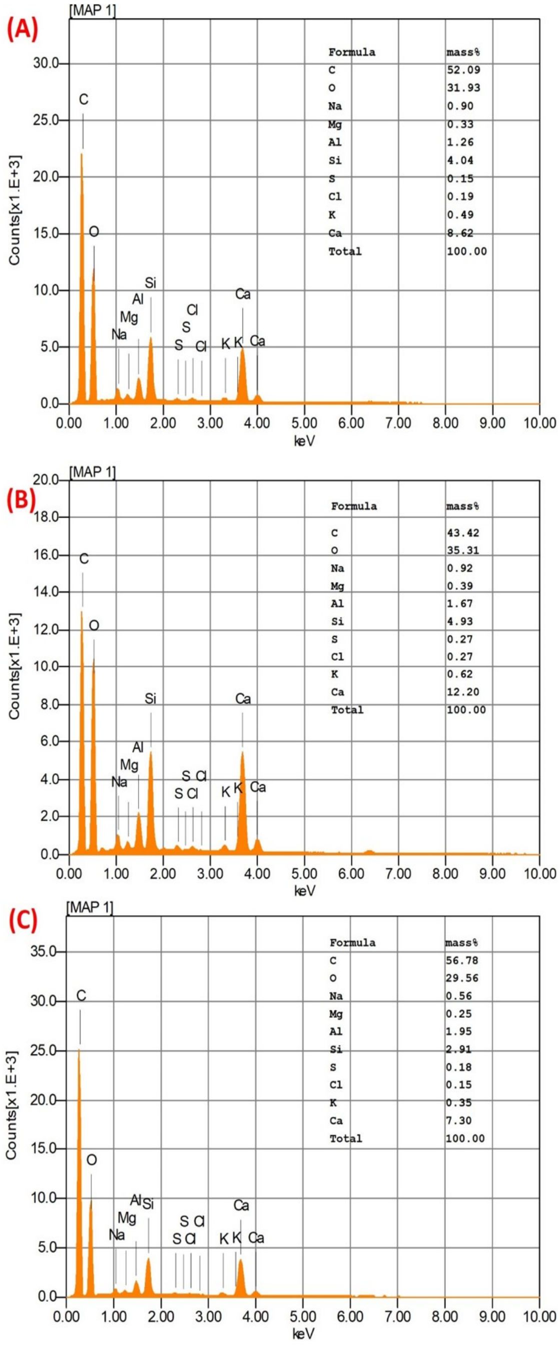

Figure 1. Spectra and semi-quantitative EDS analyses obtained from particulate matter collect at (**A**) Obispado, (**B**) Santa Catarina, and (**C**) Cadereyta monitoring stations.

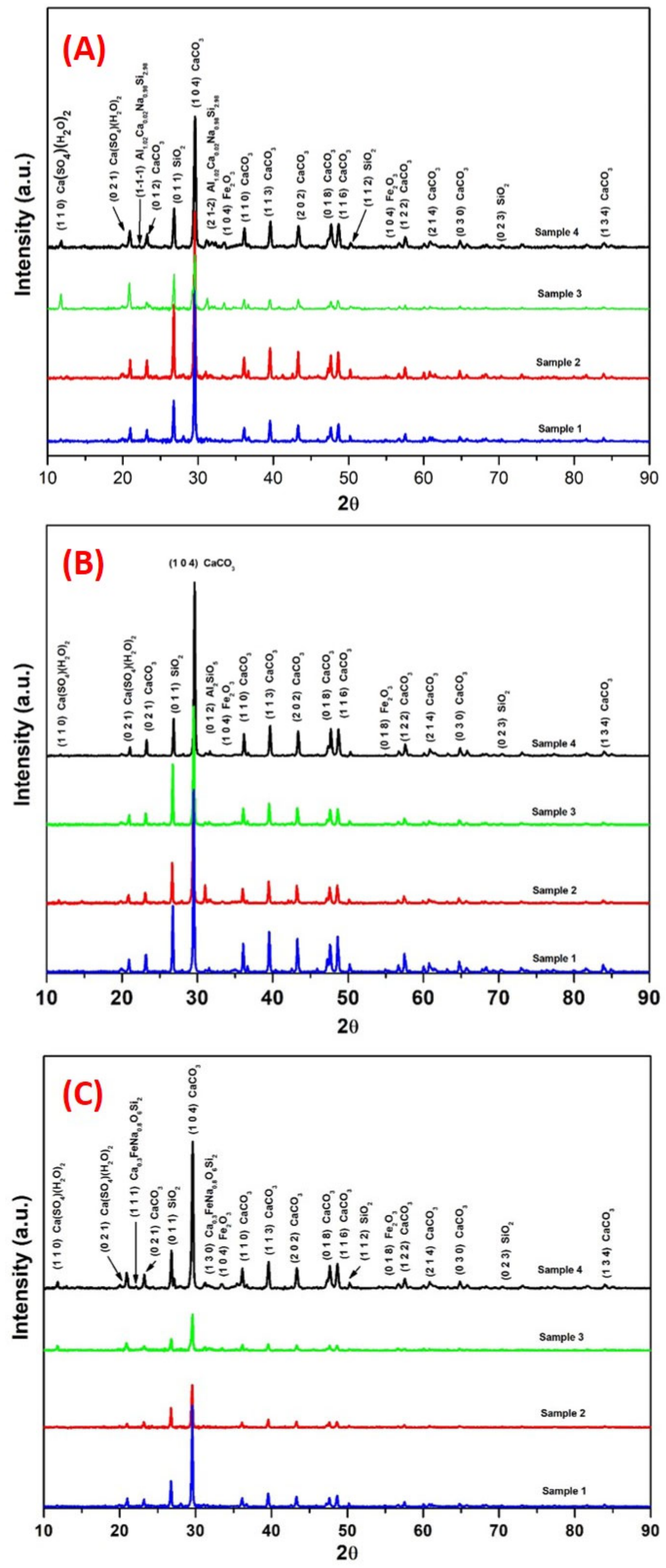

Figure 2. Diffractograms obtained from the XRD analyses on particulate matter collected at (**A**) Obispado, (**B**) Santa Catarina and (**C**) Cadereyta monitoring stations.
